# The implications of single-cell RNA-seq analysis in prostate cancer: unraveling tumor heterogeneity, therapeutic implications and pathways towards personalized therapy

**DOI:** 10.1186/s40779-024-00526-7

**Published:** 2024-04-11

**Authors:** De-Chao Feng, Wei-Zhen Zhu, Jie Wang, Deng-Xiong Li, Xu Shi, Qiao Xiong, Jia You, Ping Han, Shi Qiu, Qiang Wei, Lu Yang

**Affiliations:** 1 Department of Urology, Institute of Urology, West China Hospital, Sichuan University, Chengdu, 610041 China; 2 Division of Surgery & Interventional Science, University College London, London, WC1E 6BT UK

**Keywords:** Prostate cancer, Single-cell RNA sequencing (scRNA-seq), Tumor microenvironment, Tumor heterogeneity, Treatment resistance, Precision medicine

## Abstract

In recent years, advancements in single-cell and spatial transcriptomics, which are highly regarded developments in the current era, particularly the emerging integration of single-cell and spatiotemporal transcriptomics, have enabled a detailed molecular comprehension of the complex regulation of cell fate. The insights obtained from these methodologies are anticipated to significantly contribute to the development of personalized medicine. Currently, single-cell technology is less frequently utilized for prostate cancer compared with other types of tumors. Starting from the perspective of RNA sequencing technology, this review outlined the significance of single-cell RNA sequencing (scRNA-seq) in prostate cancer research, encompassing preclinical medicine and clinical applications. We summarize the differences between mouse and human prostate cancer as revealed by scRNA-seq studies, as well as a combination of multi-omics methods involving scRNA-seq to highlight the key molecular targets for the diagnosis, treatment, and drug resistance characteristics of prostate cancer. These studies are expected to provide novel insights for the development of immunotherapy and other innovative treatment strategies for castration-resistant prostate cancer. Furthermore, we explore the potential clinical applications stemming from other single-cell technologies in this review, paving the way for future research in precision medicine.

## Background

Prostate cancer (PCa) is one of the leading malignant tumors affecting males globally, with concerning trends in both incidence and mortality [1–3]. In China, the situation is particularly challenging, marked by advanced tumor stages, higher metastasis rates, and lower survival rates compared with some Western countries [4–6]. There is an urgent need for early diagnosis and treatment of PCa. However, the heterogeneity of PCa presents obstacles in both research and clinical management. Previous studies have shown that tumor tissue remodeling and the involvement of cancer-related fibroblasts in recurrence and metastasis underscore the complexity of PCa progression [7, 8]. Additionally, both intratumoral and transcriptional heterogeneity may contribute to the evolution of PCa [9–11]. In recent years, the introduction of single-cell RNA sequencing (scRNA-seq) has significantly advanced PCa research, offering benefits such as cell type and gene identification, correlation with clinical phenotypes, and insights into tumor heterogeneity, evolution, and ecosystem [12, 13]. Importantly, the well-known *PAM50* gene expression classifier in breast cancer has been applied to PCa research, providing relevant molecular profiles to some extent [14, 15]. From a clinical point of view, single-cell analysis enables the early diagnosis of partially radiographic-invisible PCa, predictions for therapy response, drug resistance, and prognosis, as well as guidance for the surgical process. Techniques such as multiplex immunofluorescence-based single-cell spatial imaging and single-cell analysis-assisted liquid biopsy have been instrumental in this regard [16, 17]. Single-cell analysis also addresses the challenges in precision medicine by assessing changes in molecular signatures at different stages of PCa, while molecular probes hold great potential for accurately determining surgical margins [18, 19]. Moreover, effective integration methods facilitate the generation of more meaningful results based on individual single-cell analyses. For example, Luecken et al. [20] provided a robust data integration method using a Python module and a benchmarking pipeline. All of these findings contribute to revealing the pathogenesis of the disease and providing new insights for clinical diagnosis and treatment of PCa [21].

Unfortunately, only a few studies have focused on PCa. The challenges associated with scRNA-seq include technical issues such as low RNA input, amplification bias, dropout events (false-negative signals, being particularly problematic for weakly expressed genes and rare cell populations), batch effects, cell doublets, and quality control (QC) as well as methodological hurdles like unprecedented sequencing depth, noise or biases induced during library preparation and data analysis [22]. Fortunately, multiple technical solutions have been developed [22–24], including optimized sample preparation methods, improved sequencing technologies, and specialized computational algorithms for data normalization, QC, and cell clustering. Additionally, the representativeness of the sequenced cells concerning the distribution of cells within the tissue of interest remains unclear [22]. Combining bulk and scRNA-seq for deconvolution analysis or improving cell capture throughput may offer potential solutions to these challenges. However, specific issues related to PCa- persist: 1) during the sampling stage, obtaining accurate PCa tissues is hindered by the ambiguous structure of prostate tissue, and the embedded tumor also presents a technical difficulty for spatial transcriptome sequencing; 2) at the sequencing stage, the low viability of PCa cells after tissue lysis affects specimen quality, impacting subsequent data analysis, and may be resolved by using the proper collagenase or through rigorous QC of cell suspensions; 3) other dilemmas include the requirement for fresh specimens in scRNA-seq, conflicting with the longer time needed for pathological diagnosis, while multi-sample sequencing that ignores pathological diagnosis results in high costs. Moreover, for undiagnosed patients with suspected small tumors, it is advisable to prioritize limited specimens for pathology biopsy over sequencing. The 10 × platforms (Chromium and Visium) involve tissue destruction in the workflow, thus requiring hematoxylin and eosin (H&E) and immunofluorescence (IF) labeling to be performed beforehand or on serial sections. However, the ability to detect intercellular communication processes at high-resolution (cell–cell and ligand-receptor interactions) and the capacity to precisely assign transcripts to a specific cell within a spatial context at high gene plexy are lacking [25]. The ideal method would provide high gene-plexy, high throughput, multi-modal readouts with spatial context and subcellular resolution without compromising tissue integrity, and would be suitable for both fresh-frozen and formalin-fixed paraffin embedded tissues. In addition to the aforementioned characteristics, the innovative Xenium in situ technology also includes a sizable imageable area and integrates gene expression with histological micrographs (H&E and IF staining) in the same tissue section [25]. The use of Chromium, Visium, and Xenium platforms illustrates how the integration of whole transcriptome and targeted in situ data offers highly complementary and additive biological information in human cancer, including PCa [25].

Given the significant impact of scRNA-seq in the field of cancer research, we have compiled a comprehensive review of the current evidence about the implications of this advancing technology in PCa. This effort aims to lay the groundwork for future investigations and individualized treatment strategies for patients with PCa.

## scRNA-seq and spatial transcriptome sequencing

**Fig. 1:**
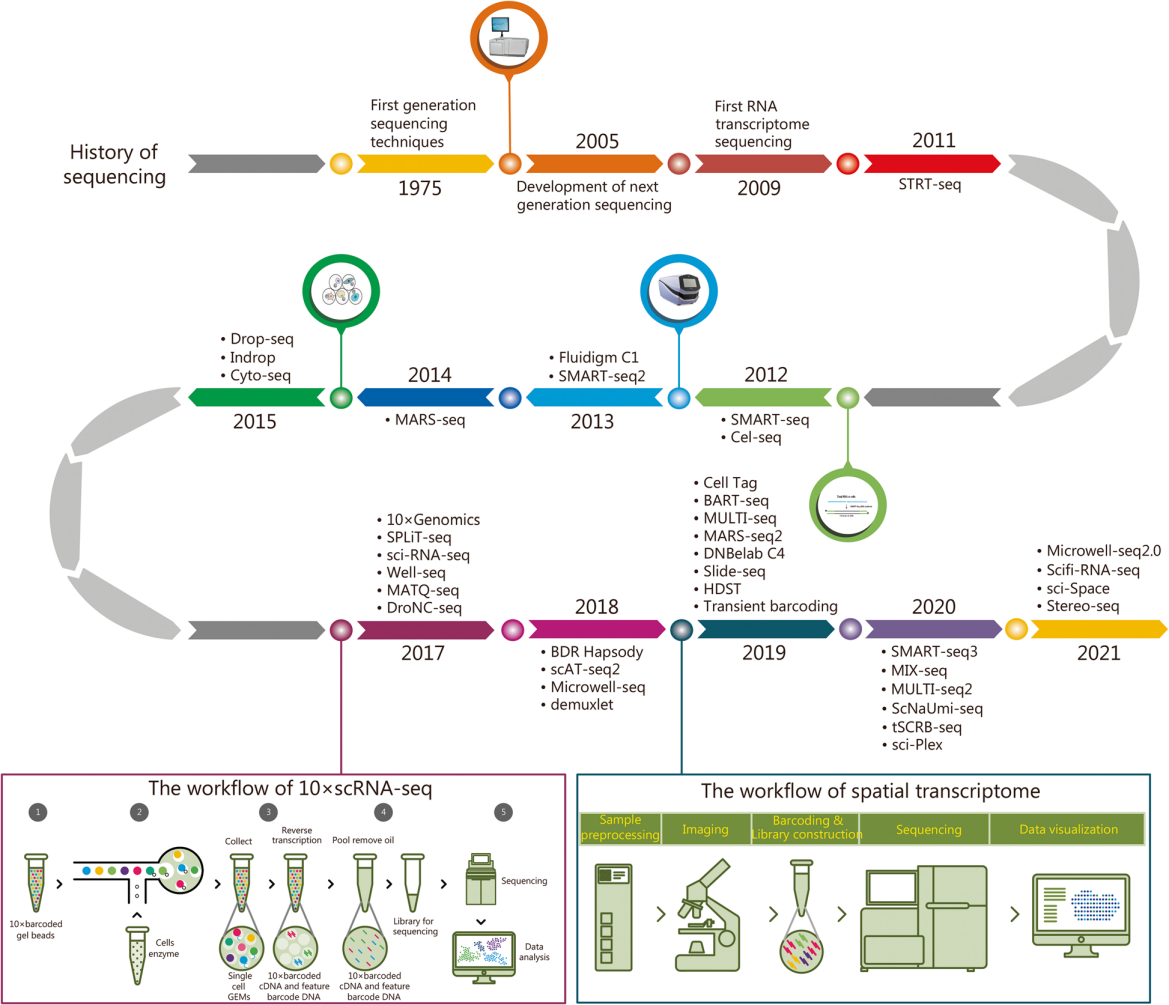
Evolution of single-cell RNA sequencing, workflow of 10 × scRNA-seq and spatial transcriptome. RNA ribonucleic acid, STRT-Seq single-cell tagged reverse transcription sequencing, MARS massively parallel RNA single cell, SMART Switching mechanism at 5’ end of the RNA transcript, Cell-seq cell expression by linear amplification and sequencing, SPLiT-Seq split-pool ligation-based transcriptome sequencing, sci-RNA-seq Single cell combinatorial indexing RNA sequencing, MATQ-Seq Multiple annealing and tailing-based quantitative single cell RNA sequencing, scAT-seq2 single-cell assay for transposase-accessible chromatin using sequencing 2, BART-seq barcode assembly for targeted sequencing, MULTI multiplexing using lipid-tagged indices for single-cell and single-nucleus RNA, HDST high-definition spatial transcriptomics, ScNaUmi-seq single-cell nanopore sequencing with unique molecular identifiers, tSCRB-seq T cell-tailored single-cell RNA barcoding and sequencing, Scifi single-cell combinatorial fluidic indexing

Currently, the most widely utilized technology for scRNA-seq is offered by 10 × Genomics. In 2017, 10 × Genomics, in partnership with the Fred Hutchinson Cancer Research Center, developed this method by integrating sample droplets and barcoding [26]. 10 × Genomics recognized the advantage in the separation and expansion of single cells, enabling the analysis of large cell populations in a short time [27]. The technique of scRNA-seq in deciphering pathological mechanisms has been extensively discussed in various diseases [28, 29]. The workflow of 10 × Genomics is shown in Fig. 1. As scRNA-seq technology continued to advance, the capacity for sequencing a greater number of cells in a single experiment and the sequencing depth have increased, while the cost of sequencing has decreased. Compared to bulk RNA-seq, the primary advantage of scRNA-seq lies in its ability to assess heterogeneity among cell populations and construct a gene expression map for each cell, facilitating the differentiation of various cell types.

### QC strategies for scRNA-seq analysis

The utilization and application of bioinformatics in the analysis of scRNA-seq have significantly transformed our comprehension of cellular heterogeneity and gene expression at a single-cell level. Depending on the care taken to design experiments, scRNA-seq can be applied to various types of samples, including PCa tissues, peripheral blood, organoids, mouse models, and even PCa cell lines subjected to different interventions. Taking the scRNA-seq raw data from the 10 × Illumina platform as an example, CellRanger is a robust tool that can decode FASTQ files into filtered data for subsequent analyses [30]. The steps involve QC for sequencing reads, preprocessing to remove low-quality bases, alignment and mapping, and cell demultiplexing as well as quantification by unique molecular identifier (UMI)-count table generation [31, 32]. After the initial processing, a new round of QC is conducted to ensure the individualization, intact, and high quality of the analyzed cell “barcodes”. Common QC indices such as “nFeature”, “nCount”, and “percent.mt” are used to assess the number of detected genes, total UMI, and the percent of mitochondrial genes, respectively. Immune cells, particularly neutrophils, typically exhibit a lower “nFeature” count compared to other cell types in humans [33]. However, cells with an extremely low “nFeature” count and high “percent.mt” may indicate low-quality cells with leaked cytoplasmic mRNA and conserved mitochondrial mRNA [34]. It should be noted that in cases where determining the minimum threshold is challenging, alternative QC indicators such as housekeeping gene expression [10], and cell metabolism levels [35] can be considered. Additionally, the Median Absolute Deviation algorithm can be employed [33]. In contrast, cells with excessive “nFeature” values may indicate the presence of cell doublets. Furthermore, the solid tissue of PCa is too hard to be digested, and the fibroblasts in its microenvironment are also apt to adhere to adjacent cells, leading to a relatively high cell doublet level. Possible solutions include limiting the maximum value for “nFeature” or employing double-cell removal tools, such as DoubletFinder [36] that show the highest accuracy in detecting cell doublets. DoubletFinder integrates predefined cell doublets with the original cell sample and performs dimensionality reduction and clustering. Theoretically, artificially simulated doublets are expected to be closer proximity to real doublets. The doublet probability for each barcode is determined by calculating the proportion of artificial doublets in the K-nearest neighbors of each cell (pANN) and sorting the values accordingly. Furthermore, the Poisson distribution can be used to estimate the number of doublets in each sample. It is important to consider the pervasive presence of environmental RNA contamination throughout the entire sequencing process, from cell suspension, and sorting to reagent selection, and even potential operational errors, which may introduce contaminants into the sequencing pool. Recently developed methods such as DecontX [37] and SoupX [38] effectively address these issues, thereby reducing noise in downstream analyses. While recent single-cell studies have not adjusted the QC strategy according to samples or data situation [33], it is recommended to adopt flexible QC cutoff values based on different situations. Additionally, a later QC strategy after primary data processing and cell type annotation, followed by reanalysis of the data with high-quality cells, may yield improved results.

### Basic processes and tools for scRNA-seq analysis

The matrix derived from processing raw data may not accurately represent the absolute quantity of mRNA molecules, due to variability in total UMI counts per cell, influenced by numerous technical and biological factors. Factors such as RNA capture efficiency, reverse transcription, cDNA amplification, sequencing depth during library preparation, cell sizes and cell cycles contribute to differences in cell expression profiles between cells and samples. To address this issue, scRNA-seq often employs “Normalization” to eliminate technique-driven differences and enhance data comparability and accuracy while preserving real biological variations among cells [39]. “Scaling” is another concept related to “Normalization”, involving the linear transformation of data to fall within a specific range to eliminate the impact of scale differences on analysis. Overexpression of certain genes, such as those related to cell cycle, cell stress, and mitochondria, can obscure true biological alterations, which can be addressed through linear regression during the “Scaling” process. Common R packages for scRNA-seq analysis, such as “Seurat” and “SingleCellExperiment” provide similar functions to get a normalized (log-transformed) or scaled matrix. For improved variance stabilization, the Seurat team has recently introduced SCTransform, a method that employs regularized negative binomial regression for normalizing and stabilizing variance in scRNA-seq data [40]. It is important to note that these procedures can be directly applied to single-cell sample data, but for the analysis of multiple samples, data integration and batch effect removal are usually necessary. Batch effect refers to problems arising from non-biological differences, such as experiment time and sample heterogeneity. Alternative methods include: 1) “Seurat”, which involves the integration and batch correction of scRNA-seq datasets through techniques such as Seurat integration and canonical correlation analysis [41]; 2) “Harmony”, which enables batch effect removal and integration of scRNA-seq datasets by adjusting for technical variation [42]; 3) “Scanorama [43]”, “FastMNN” [44], and other possible algorithms [45].

Although a large number of low-quality cells have been removed during the QC procedure, more than 20,000 genes are typically mapped and annotated in the complete data from a study. Not all genes provide valuable biological information, and analyzing all genes would be inefficient in terms of time and resources. Hence, the approach of “Feature Selection” from the field of machine learning and statistics is used to select a subset of genes (often ranging from 2000 to 5000) for subsequent unsupervised analysis [34, 46]. A commonly employed method involves the selection of highly variable genes (HVGs) [47] and the use of Seurat to identify HVGs based on their variance. It is important to note that in scRNA-seq, the distribution of cells expressing thousands of genes exists in a high-dimension space, and efforts are made to render it comparable and visible in a low-dimension space.

Seurat works on the selected HVGs to implement dimensionality reduction and clustering techniques such as principal component analysis, t-distributed Stochastic Neighbor Embedding [48], and Uniform Manifold Approximation and Projection [49], as well as graph-based clustering algorithms. Another R package, “SingleCellExperiment”, offers infrastructure and methods for storing, manipulating, and analyzing single-cell genomics data, including dimensionality reduction and clustering. Subsequently, annotating the acquired clusters may be a crucial step, with manual annotation being more accurate but largely relying on existing biological knowledge, where differential analysis plays a role. The “Seurat” package functions “FindMarkers/FindAllMarkers” are convenient tools for identifying cluster-specific genes for improved annotation. Additionally, “MAST” is another R package specialized for differential expression analysis in scRNA-seq data, accommodating zero inflation and modeling cell-specific parameters [50]. For unfamiliar cell types, the combination of automatic annotation with manual annotation may be a more ideal solution, with SingleR [51] and CellTypist [52] being widely-used automatic annotation tools.

### Advanced processes and tools for scRNA-seq analysis

With precise and thorough annotation, subsequent methods of functional analysis, cell communication, and trajectory inference analysis in cell development become applicable. Notable functional analyses include gene ontology, gene set enrichment analysis, and gene set variation analysis that can be achieved by “clusterProfiler” [53], “GSEABase” [54], and various websites. Moreover, advanced tools for cell communication analysis, such as CellPhoneDB [55] and CellChat [56], provide insights into signaling pathways, ligand-receptor interactions, and cellular crosstalk, playing crucial roles in the analysis of various biological processes and disease mechanisms.

Importantly, the necessity and value of uncovering the dynamics and relationships within cellular processes should be considered during bioinformatic analysis for scRNA-seq data. Trajectory analysis is a widely used method for inferring cell transition, differentiation processes, cell fate decisions, or disease progression based on gene expression similarities. It includes two parts: pseudotime analysis and trajectory inference. Pseudotime analysis involves inferring the temporal ordering of cells along developmental trajectories or biological processes, often using algorithms like “Monocle” [57] or “Slingshot” [58]. While trajectory inference reconstructs cellular trajectories to visualize dynamic processes, such as cell differentiation or disease progression, using algorithms like “Wishbone” or “Tools for Single Cell Analysis (TSCAN)” [59, 60]. However, lineage-tree-based analysis may be a more suitable and direct method that focuses on constructing a tree-like representation of the cellular lineage or differentiation hierarchy by measuring mitotic histories to capture the hierarchical relationships between cells and the branching events during development [61]. Both trajectory and lineage-tree-based analyses operate at the individual cell level, relying on computational algorithms and statistical methods to capture and analyze the temporal dynamics of cellular processes, providing insights into cellular development, differentiation, or disease progression, and offering a better interpretation of the underlying mechanisms and regulatory networks driving cellular processes. However, the unique power of lineage-tree-based analysis lies in its ability to describe a continuum or a ‘landscape’ of cell states and transition trajectories between different states during cell redifferentiation at a high resolution by employing strategies for single-cell state manifold reconstruction and lineage barcoding [61].

Decades ago, the understanding of cell function was primarily based on some simple characteristics such as location, morphology, staining, and basic chemical or physical properties. The advent of various sequencing technologies has revolutionized our understanding of cells and changed the modes of studying cells, with scRNA-seq emerging as a prominent technique. Supplementary materials of this review show the evolution history of scRNA-seq technology. Based on large scRNA-seq datasets, lineage-tree based analysis can be employed to characterize the specific gene expression continuum of novel cell states or cellular developmental transitions compared with transient information captured by conventional analysis. Multiple algorithms have been proposed to deduce, visualize, and even predict the cell dynamics and hierarchal states, directly transforming a manifold to a tree-like structure. Moreover, the precise determination of a cell’s lineage history relies on barcode-sequencing. Specifically, researchers introduce individual cell-specific DNA barcodes into different cells to facilitate the tracing of changes in the targeted whole-genome or mitochondrial-genome sequencing data, thereby constructing a lineage phylogeny as each cycle of mitosis occurs [61]. Notably, the DNA barcodes permanently alter the genome of a single cell, making it easy to identify its progeny cells that inherit this alteration. The high-throughput recording and measurement of DNA barcoding events makes it possible to trace thousands of different cloning units in parallel, and the accumulation of barcodes contributes to the phylogenetic reconstruction of cell lineage trees. Three types of barcodes are employed. First, the integration of an exogenous DNA barcode library via transposase using transposon-based barcoding approaches (TracerSeq [62] and CellTagging [63, 64]). Second, in vivo recombination of transgenic DNA cassettes employing PolyLox [65, 66]. Third, in vivo accumulative insertions and/or deletions of random error during CRISPR-Cas9 editing of genomic target sites that is innovative and employs synthetic target arrays for lineage tracing (GESTALT) [67], single-cell GESTALT (scGESTALT) [68, 69], ScarTrace [70], lineage tracing by nuclease-activated editing of ubiquitous sequences (LINNAEUS) [71], CRISPR Array Repair LINeage tracing (CARLIN) [72] and so on. In an informative report by Montoro et al. [73], it was confirmed that Krt5-CreER-marked basal cells regenerated all epithelial cell types of the airway tissue through classical genetic recombination-based lineage-tracing methods and scRNA-seq. Unfortunately, there are currently no studies combining scRNA-seq with lineage analysis on PCa, and lineage tracing methods to identify the progenitor cells of PCa may be a promising direction for future research.

### Integration of scRNA-seq with spatial transcriptomics

The process of breaking down tissue with enzymes to obtain individual cells results in the loss of spatial information about the cells within the tissue. This can lead to changes in gene expression and the loss of valuable information. In 2016, a new technique called spatial transcriptome was developed to address this issue, allowing for the retention of spatial location information while obtaining gene expression data. This technique, which included Slide-seq, HDST, and sci-Space [74–77], was recognized as the “method of the year” by *Nature Methods* in 2020 [78]. The workflow is depicted in Fig. 1.

Combining spatial transcriptome methodology with scRNA-seq helps the integration of spatial location information with the multimodal features of cell populations. In 2021, the Beijing Genomics Institute proposed the concept of a spatiotemporal transcriptome and developed a Stereo-seq, which added a temporal dimension to the spatial transcriptome information to observe the transcriptomic changes during cell development [79]. This breakthrough technique achieved subcellular-level resolution and a centimeter-sized imageable field, providing a greater field of view and higher resolution than other mainstream technologies [79].

Figure 1 illustrates the timeline of scRNA-seq development. Hirz et al. [80] combined scRNA-seq with spatial transcriptomic analysis to improve the understanding of PCa. This combination provided a detailed resource on the PCa microenvironment and tumor-stromal cell interactions, shedding light on the causes of a suppressive tumor microenvironment [80]. They also identified Hillock and Club cells as progenitor cells and deduced a robust prostate tumor gene signature. This thorough examination of the cellular and molecular landscape of PCa will help identify vulnerable areas for therapeutic intervention. Moreover, Tuong et al. [81] employed scRNA-seq to reveal a unique zinc transporter-expressing prostate-specific macrophage population called “MAC-MT”, which may enhance immune responses and—counteract fluctuations in zinc concentrations associated with PCa. Spatial transcriptomics provided valuable validation in this context. The work of Barkley et al. [82] established a framework for studying how cancer cell states interact with the tumor microenvironment to form organized systems capable of immune evasion, drug resistance, and metastasis by pan-cancer scRNA-seq and spatial transcriptomics analysis. The characteristics of the three transcriptome patterns described above are presented in Table 1.

**Table 1:** Comparisons among bulk, single-cell, and spatial transcriptomes

Items	Bulk transcriptome	Single-cell transcriptome	Spatial transcriptome
Analytic object	Tissue	Cell	Tissue section
Tumor cell region	Not applicable	Presumed by the algorithm	Identified directly on sections
Dimensionality reduction	Not applicable	Principle component analysis (PCA), t-distributed stochastic neighbor embedding (t-SNE), or uniform manifold approximation and projection (UMAP)	Principle component analysis (PCA), t-distributed stochastic neighbor embedding (t-SNE), or uniform manifold approximation and projection (UMAP)
Cluster	Not applicable	k-means, louvain or hierarchical clustering based on cell type	k-means, louvain or hierarchical clustering based on different functional areas
Differential expression analysis	For different tissues	For cell clusters	For spatial location
Enrichment analysis	Gene function differences between different tissues	Biological functional differences between the cell clusters	Gene function differences at different spatial locations
Advantage	The price is low	Cell resolution	The spatial location information is retained
Limitation	The result is the average gene expression within the tissue, and the precision is low	Missing the spatial location information	Technically, the resolution is lower than single-cell transcriptome in most cases

## scRNA-seq analysis in PCa research

### scRNA-seq analysis reveals similarities and differences of prostate components in mouse model and human

**Fig. 2:**
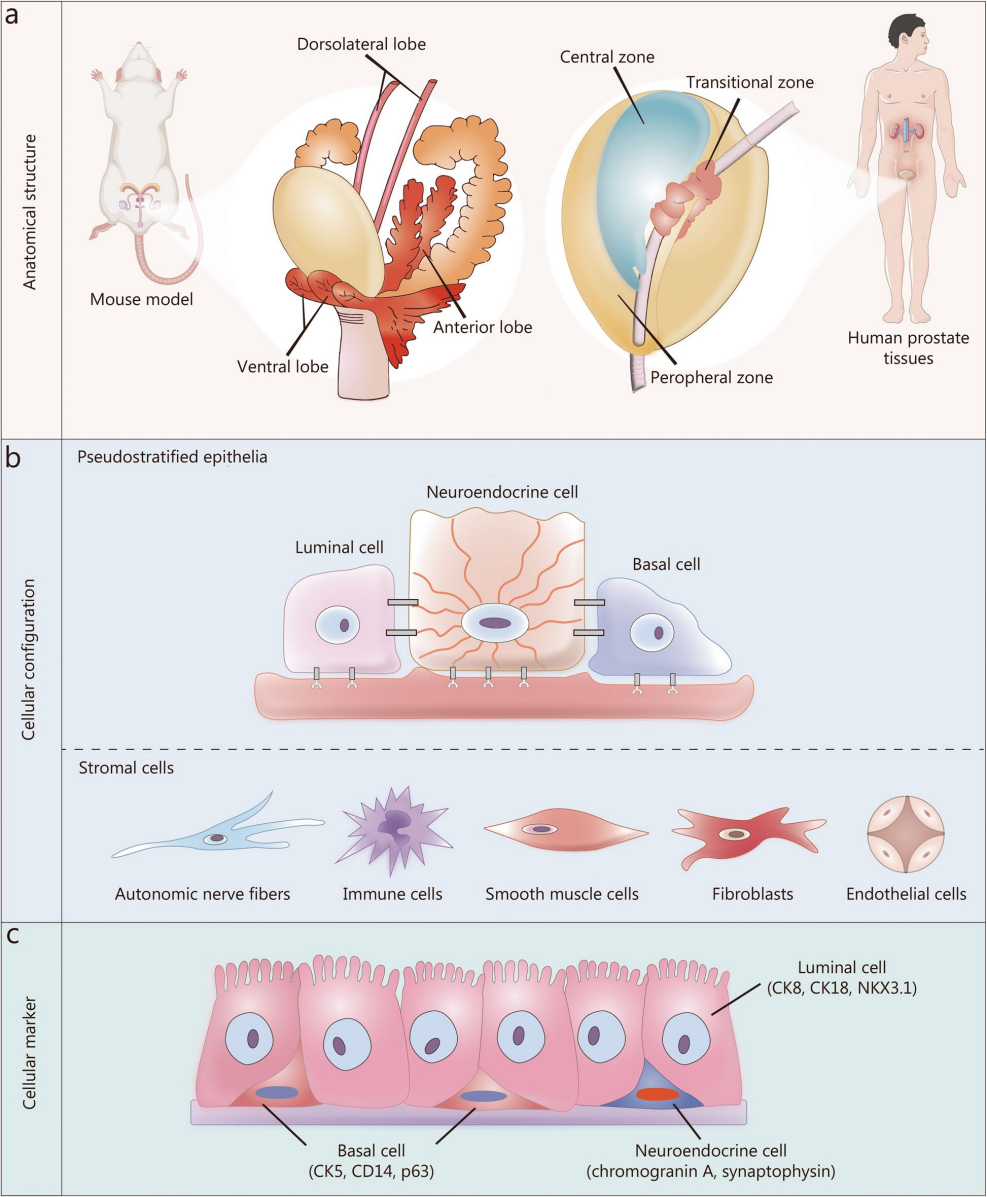
Comparison of mouse models and human prostate tissues at anatomical structure, cellular configuration and cellular marker levels. **a** The mouse prostate can be divided into: anterior lobe, ventral lobe and dorsolateral lobe and the human prostate can be divided into: peripheral zone, transitional zone and central zone. **b** The prostates of both mice and humans are composed of pseudostratified epithelia and stromal cells. Pseudostratified epithelium consists of three types of cells: luminal cells, neuroendocrine cells, and basal cells. Stromal cells are further categorized into autonomic nerve fibers, immune cells, smooth muscle cells, fibroblasts, and endothelial cells. **c** The cellular markers of basal cell contain CK5, CK14 and p63. The cellular markers of luminal cell contain CK8, CK18 and NKX3.1. The cellular markers of neuroendocrine cell contain chromogranin A and synaptophysin. CK cytokeratin

The mouse prostate model is a commonly used animal model in scientific research for studying prostate-related diseases and testing the efficacy of treatments. It can be employed to investigate the pathogenesis, pathophysiological changes, and assess the effectiveness of therapies for conditions like prostate cancer and benign prostatic hyperplasia. However, there are limitations compared with human disease models, such as anatomical and cellular marker differences, requiring caution in interpreting experimental results. From anatomical perspective, PCa and benign prostatic hyperplasia develop in different zones of the prostate [83, 84]. In a young prostate, the peripheral zone is larger, while the transitional zone is small and has a regular structure that becomes larger and irregular with aging [85]. The study conducted by Yan et al. [86] utilized scRNA-seq technology to observe that older prostate tissues with more Trefoil factor 3 -positive cells in the peripheral zone are more prone to malignant transformation. However, when using experimental mouse models to explore human prostate-related diseases, it is important to note that the structures of the mouse prostate, including the anterior lobe, ventral lobe, and dorsolateral lobe, consisting of dorsal and lateral lobes, are significantly different from those of humans [87]. Conversely, human and mouse prostate cells have a similar composition at the cellular configuration level. The prostates of both mice and humans are composed of pseudostratified epithelia and stromal cells. Pseudostratified epithelium consists of three types of cells: luminal cells, neuroendocrine cells, and basal cells. Stromal cells are further categorized into autonomic nerve fibers, immune cells, smooth muscle cells, fibroblasts, and endothelial cells. Baures et al. [88] utilized scRNA-seq to demonstrate that the LSC^med^ cells from mouse prostate, which are molecularly equivalent to luminal progenitor cells, closely resemble Club and Hillock cells in the human prostate. From the perspective of cellular marker, each lobe exhibits distinct transcriptomic differences. In mice, ‘prostate’ fibroblasts are marked by expression of complement C3, early B-cell factor 1 transcription factor 1, glutathione peroxidase 3, sulfotransferase family 1E member 1 and insulin like growth factor 1, while ‘ductal’ fibroblasts are characterized by the expression of Wnt2, Rorb, Wif1, Ifitm1 and Srd5a2 [89]. In contrast, podoplanin, decorin, fibroblast activation protein α, and collagen type I α 1 chain appear to be reliable markers for fibroblast in the human prostate [90]. Luminal cells in FVB/NJ mice exhibited specific markers for different lobes and an increased presence of proto-oncogene-related targets [91]. According to the 10 × scRNA-seq data of Crowley et al. [92], Transglutaminase 4 was identified as the marker for luminal cells in AP and DP, microseminoprotein β for LP, and protein phosphatase 1 regulatory inhibitor subunit 1B for all lobes. Henry et al. [90] performed flow cytometry and scRNA-seq on approximately 98,000 cells from different anatomical regions of the young adult human prostate and prostatic urethra, and they found CD200 to be more effective marker for endothelium than CD31, a widely used but inefficient marker for endothelial cells in the prostate. Human ductal luminal cells, expressing keratin 7 and retinoic acid receptor responder 1, were most closely related to proximal luminal cells in mice, while the acinar luminal cells, expressing microseminoprotein β and membrane metalloendopeptidase, were most closely related to luminal cells of LP-specific followed by VP-specific tissue [92]. Microarray profiling confirmed that the dorsolateral lobe was most similar to the peripheral zone in the human prostate [93]. Additionally, there are several similar cellular markers in both humans and mice. For example, cytokeratin (CK) 8, CK18, and NK3 homeobox 1 as well as CK5, CK14 and tumor protein p63 are expressed in both mouse and human luminal cells and used to label luminal cells and basal cells in both species [94]. Therefore, the significance of using mouse models to study the initiation, heterogeneity, and development of PCa from different cell lineages should be considered. For instance, Guo et al. [95] defined a unique luminal cell subtype as the cellular progenitor of PCa by profiling 35,129 mouse prostate cells and 11,374 human prostate cells through scRNA-seq. They also confirmed the presence of luminal-C cells in the human prostate and confirmed their potential role as PCa progenitor cells. The markers for different luminal cells (luminal-A, luminal-B, and luminal-C) found in mice, including tumor associated calcium signal transducer 2, prostate stem cell antigen, and keratin 4, were also suitable for human prostate tissue [95]. Figure 2 summarizes the similarities and differences between anatomical structure, cellular configuration, and cellular marker of human and mouse prostate.

As a result, findings from analyzing individual cells in a mouse model can sometimes be applied to human prostate cancer. This is supported by recent studies using scRNA-seq in wild-type mouse prostates, which have revealed the adaptability of luminal cells after castration, a phenomenon also observed in human benign prostatic hyperplasia [96, 97]. In addition, studies have found similar differentiated stem-like luminal subpopulations in human prostate, organoids and mouse models using scRNA-seq, showing proliferative activity after androgen deprivation [96, 98–100]. Obviously, cancer cells inhabit natural stem cell-like niches within normal tissue [101]. Untreated or surviving PCa cells after varied therapies can exploit the stem-like phenotype of luminal cells to drive tumor progression, recurrence, or castration resistance. Hence, in situ or distant metastasis of PCa may be associated with luminal cell subtypes. Similarly, the scRNA-seq study of Baures et al. [88] demonstrated that LSC^med^-like luminal progenitor cells in mice were intrinsically castration-tolerant in both healthy and cancerous tissues, as were cells in the human prostate, particularly those associated with PCa initiation [102]. Moreover, Han et al. [103] utilized multi-omics data from more than 100,000 cells from genetically engineered PCa mouse samples to reveal that FOXA2 orchestrated the transition from adenocarcinoma to neuroendocrine lineage. Overall single-cell analysis in mouse models sheds light on the mechanisms underlying the development and progression of human PCa, suggesting targeting normal luminal cells with new therapies could be beneficial in preventing tumor progression and improving prognosis during the treatment of PCa.

### scRNA-seq analysis clarifies PCa tumor heterogeneity

PCa originates in luminal and basal cells [104–106]. scRNA-seq data has shown that different types of luminal cells are associated with varying degrees of malignancy and clinical outcomes in PCa. Ma et al. [107] identified highly malignant type 1 luminal cells related to early progression of PCa, type 3 luminal cells associated with migration, and less malignant type 2 luminal cells linked to poor prognosis. They also found that specific genes such as α-methylacyl-CoA racemase, prostate cancer antigen 3, and hepsin (HPN) have significant diagnostic value [107–109]. Additionally, scRNA-seq results may help categorize high-risk PCa subtypes into more representative and common populations [110]. Chen et al. [10] identified a unique cluster of luminal cells termed “Cell cycle” that showed high expression of high-risk PCa signatures (luminal B, hypoxia, and prostate cancer subtype 1 [111, 112]). Another study using scRNA-seq analysis of human prostate samples identified specific luminal and fibroblast-derived PCa progenitors and potential genes and cell functions, including a link between cellular senescence and PCa development [113]. Furthermore, it has been reported that drug resistance in PCa is derived from an epithelial population with a mixed luminal-basal phenotype, as supported by single-cell data from both mouse models and human patients [114]. These studies indicate the potential of scRNA-seq in identifying populations at common risk for PCa.

### scRNA-seq analysis aids in understanding the PCa tumor microenvironment

**Fig. 3:**
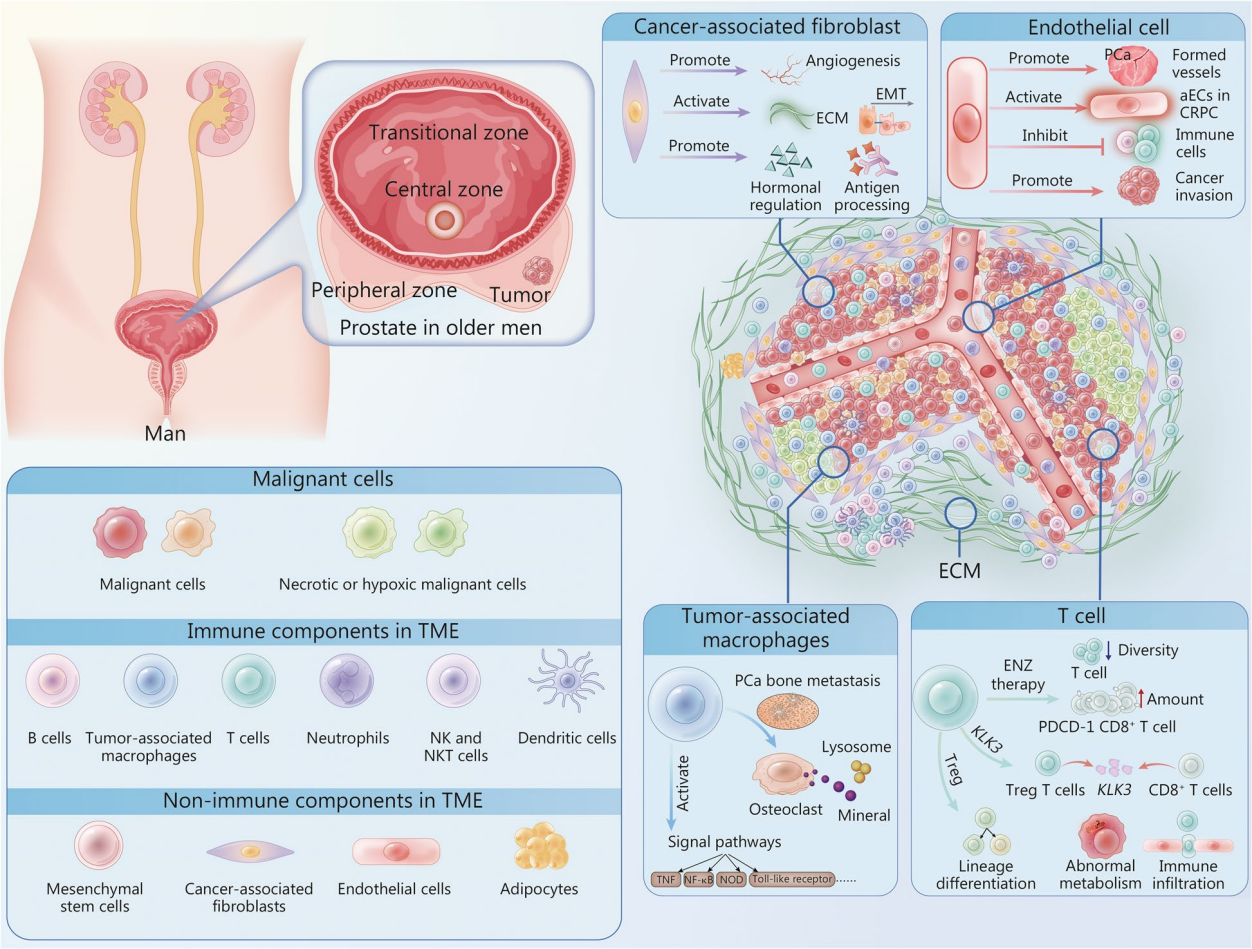
The tumor microenvironment of prostate cancer. In the PCa TME, both immune and non-immune components played crucial roles in the PCa initiation, development, and progression. For example, Tregs were associated with lineage differentiation and immune infiltration, and TAMs exhibited unexpected OC-like features, potentially promoting bone metastasis. Additionally, CAFs contributed to extracellular matrix activation and epithelial-mesenchymal transition (EMT). Besides, abnormal aECs enriched in CRPC inhibited immune responses and enhanced tumor invasion. PCa prostate cancer, TAM tumor-associated macrophages, ECM extracellular matrix, EMT epithelial-mesenchymal transition, CRPC castration-resistant prostate cancer, ENZ enzalutamide, KLK3 kallikrein 3, TME tumor microenvironment, TNF tumor necrosis factor, NF-κB nuclear factor-kappa B, NOD nucleotide binding oligomerization domain, PDCD-1 programmed cell death-1

Both immune and non-immune components in the tumor microenvironment (TME) participate in PCa initiation, development, and progression. Currently, available studies on single-cell analysis have revealed the special roles of T cells, tumor associated macrophages (TAMs), cancer associated fibroblasts (CAFs), and activated endothelial cells (aECs) in the TME. Wu et al. [115] confirmed that cryopreservation did not affect PCa heterogeneity for scRNA-seq, thus broadening the usage scenarios and research conditions of scRNA-seq. Wong et al. [116], utilizing scRNA-seq and TCR sequencing to study, found that antitumorigenic immune cells were suppressed in the TME while protumorigenic immune cells were enriched in aggressive cribriform PCa [116]. Using Metabolic Assay-Chip (MA-Chip) and scRNA-seq analysis after liquid biopsy, Rivello et al. [117] found that non-malignant stromal cells in PCa cells exhibited abnormally high metabolic activity and expressed genes related to PCa progression pathways, including angiogenesis, cell cycle activation, and oncogenic signal transduction. As for TAMs, in addition to some expected pathways related to macrophage activation and function (such as TNF, NK-κB, and NOD pathways), Chen et al. [10] found that TAMs surprisingly showed osteoclast-like features in PCa samples sequenced by scRNA-seq, which may promote bone metastasis in PCa. The appearance of TAMS was also related to the exhaustion of distinct T cell subsets in metastatic PCa [118]. Multiple studies supported this finding: Wong et al. [116] conducted scRNA-seq to demonstrate that decreased T cell numbers and increased T cell dysfunction were prominent features of the TME in aggressive cribriform PCa. Hirz et al. [80] linked immunosuppressive myeloid and T-cell exhaustion to PCa metastasis using a combined dataset of single-cell and spatial transcriptomic analyses. Interestingly, *KLK3*, the gene encoding prostate-specific antigen (PSA) [119], is expressed in almost all T cells in the TME, and the extracellular vesicles of PCa cells induced transcriptomic changes that promoted micrometastasis, further confirming the interactions between tumor cells and the TME [10]. In a study involving several patients with metastatic castration-resistant PCa (mCRPC), scRNA-seq results suggested decreased T cell diversity and increased dysfunctional programmed cell death-1 (PDCD-1, encoding PD-1 [120])-expressing CD8^+^ T cells [also known as regulatory T cells (T reg cells)] after enzalutamide therapy. Treg cells were associated with lineage differentiation and subsequent cellular metabolism, cellular communication, and immune infiltration of PCa. This indicates an elevated abnormal antitumor immune response and enzalutamide resistance [121]. Not surprisingly, single-cell analysis also assists in elucidating the regulatory network between the immune and non-immune components in the TME. For example, cancer-associated fibroblasts (CAFs) are involved in angiogenesis, extracellular matrix (ECM) activation and an epithelial-mesenchymal transition (EMT) [113]. Interestingly, fibroblasts are related to antigen processing and presentation as well as hormonal regulation in normal prostates [113], and it is reasonable to speculate that CAFs may show similar features. scRNA-seq analysis showed that a heterogeneous CAF population surrounded more aggressive PCa foci, possibly due to its immunosuppressive and poor prognosis-related proteins [116]. Furthermore, aECs are enriched in CRPC, which suppresses immune activation and promotes cancer invasion [10]. The complex network of cell–cell interactions in the TME is summarized in Fig. 3.

These changes in the transcriptome and consequently in transcription and translation may occur before alterations in cellular activities, and TME remodeling is also accompanied by changes in the transcriptome. This technology will be useful for identifying genes that contribute to PCa development and its related biological processes, further clarifying the complex mechanisms of PCa etiology, and fundamentally preventing the occurrence of PCa. Therefore, using scRNA-seq to detect these changes as early as possible is essential to continue subsequent studies and further interventions.

## scRNA-seq analysis in PCa for clinical applications

### scRNA-seq analysis is a potential assistance to improve PCa diagnostic accuracy

Although pathological biopsy is the gold standard for diagnosing PCa, its effectiveness is limited due to its invasive nature and poor accuracy in puncturing. Additionally, PSA, another sensitive diagnostic biomarker, (can sometimes be affected by benign prostatic hyperplasia and prostatitis. Imaging tests, like prostate multiparametric MRI, are also restricted by certain confounding factors and variations in how physicians interpret the results. In recent years, a new technique termed “liquid biopsy” has been developed [17, 122]. This technique involves the use of circulating tumor cells (CTCs) and offers advantages such as non-invasiveness, ease of administration, high repeatability, and the ability to overcome issues related to tumor heterogeneity. A single-cell technique called apheresis addresses the challenge of insufficient CTC in blood, allowing for further exploration of PCa heterogeneity at the genomic or transcriptomic level [123]. In contrast, scRNA-seq improves the diagnostic potential of epithelial cell adhesion molecule (EpCAM)-negative cells in biopsies. EpCAM has long been a common and typical prognostic marker for PCa CTCs [124]. In a study by Rivello et al. [117], it was found that genes significantly expressed in EpCAM-negative cells in the blood, such as androgen receptor (AR), erythroblast transformation-specific-related gene, homeobox B2, kallikrein-3 and prostate cancer associated 3, helped in identifying either EpCAM-negative CTCs or EpCAM-negative circulating stromal cells.

### scRNA-seq analysis provides novel personalized therapy possibilities for PCa patients

Androgen deprivation therapy (ADT) has been the main treatment for advanced PCa for decades. Although patients with metastatic PCa who are not eligible for radical surgery or radiotherapy exhibit favorable initial responses to ADT, a large proportion of them will eventually progress to CRPC [125]. It has been believed that CRPC develops as result of ADT selection. However, a study has shown that CRPC may not solely originate from acquired evolutionary selection during ADT, but may also be associated with highly adaptable CRPC-like cells in the early stages of PCa. These cells continuous to clone and amplify throughout the disease progression [126]. The study applied scRNA-seq to analyze around 23,000 PCa epithelial cells and identified a small proportion of highly adaptable CRPC-like cells [126]. Moreover, they pinpointed 13 key transcription regulators, such as SOX2, AR, and FOXA1 that modulate the progression towards small cell neuroendocrine carcinoma (NEPC) or CRPC [126], providing insights into effective therapeutic strategies. Another study confirmed that a fraction of luminal cells with stem cell-like potential persisted after castration [96]. Therefore, detecting pre-existing CRPC-like cells in the early stages of untreated PCa using scRNA-seq and applying this information for early interventions could offer a novel approach to CRPC therapy.

Additionally, combining the varied findings from different single-cell analyses could help in developing effective therapeutic strategies. For example, Qiu et al. [127] integrated scRNA-seq with chromatin immunoprecipitation followed by sequencing (ChIP-seq) and found that the overexpression of *c-Myc* hindered the typical *AR* transcriptional process, contributing to the progression of PCa. Considering that AR-targeted therapy-resistant PCa cells often still express Ars [128, 129], targeting *c-Myc* to restore AR activity may be an ideal strategy for managing PCa that has progressed to CRPC or even mCRPC, and many small-molecule inhibitors have been developed for this purpose in recent years [130]. From a dietary perspective, reduced saturated fat intake inhibited *c-Myc* transcription [131]. Tang et al. [132] identified activator protein-1 as a potential therapeutic target for AR-negative CRPC by integrating data from ATAC-seq, ChIP-seq, and scRNA-seq. Furthermore, He et al. [121] analyzed the single-cell transcriptomes of patients with advanced PCa, covering various common metastatic sites, including bone, lymph nodes, and liver. Their findings showed that genes in the transforming growth factor pathway could be potential therapeutic targets for mCRPC, along with multiple AR isoforms and their specific inhibitors. They also identified three genes expressed in NEPC (homeobox B5, homeobox B6, and nuclear receptor subfamily 1 group D member 2) as candidates for functional investigation due to their potential role in mCRPC [121]. The acquisition of these phenotypes by NEPC, as a lethal subtype of CRPC, remains a topic of debate. Wang et al. [133] performed scRNA-seq profiling to establish a transcriptomic map of NEPC transdifferentiation and identified the key genes involved in cell differentiation and terminal outcome. Targeting these genes is important for drug development and early intervention against NEPC transformation. It is also remarkable that the majority of treatments may affect tumor heterogeneity and the subsequent response to some extent. Therefore, it is critical to clarify the initial features of PCa, its developmental status, and subclonal evolution. Ge et al. [134] provided valuable insights by performing scRNA-seq for untreated PCa patients, revealing that non-hereditary developmental heterogeneity could overlay tumor subclonal evolution, and that transcriptional heterogeneity may also play a role in PCa progression and evolution, depending on the characteristics of mapping developmental states onto tumor subclonal evolution of scRNA-seq.

Therapeutic opportunities for PCa lie within the targets present in TME. Although immunotherapy has made great progress for multiple cancer types, its effectiveness in PCa is limited [135–137], possibly due to the low infiltration of tumor-associated T lymphocytes into the TME [138]. Peng et al. [139] identified prostaglandin E2 receptor 4 (PTGER4, also known as EP4) as a universal marker of T-cell exhaustion and immune microenvironment. Their research using scRNA-seq and pseudotime analysis led to the development of a novel antagonist YY001, which combined with an anti-PD-1 antibody, effectively weakened immune inhibition, reversed resistance to immunotherapy, and resulted in long-term survival and significant tumor regression in PCa patients, particularly those with CRPC [139]. Additionally, TAM contributes to chemotherapy resistance in cancer cells. Decreasing the number of TAMs has been shown to improve the effectiveness of docetaxel in CRPC patients [140]. Masetti et al. [141] analyzed the transcriptional landscape of human PCa at a single-cell level and identified a specific subset of TAM associated with poor prognosis, as well as the characteristic dysregulation target of lipid metabolism “Marco”. Consequently, blocking “Marco” successfully inhibited tumor growth and invasion, and simultaneously improved the efficacy of chemotherapy in mouse models, providing a new strategy for clinical decision-making [141]. Dietary control may offer multiple benefits for PCa patients, as lipid metabolism is also involved in the progression of CRPC. A recent proteomics study using single-cell mass cytometry analysis and protein quantification of 1,600,000 cells suggested TAMs as potential therapeutic targets [142]. scRNA-seq analysis has further revealed that CAFs are closely related to TME immunosuppression, highlighting their potential as targets for PCa immunotherapy [116]. Antiangiogenic agents represent another promising strategy for targeting TME. Recently, Heidegger et al. [143] confirmed that C-X-C chemokine ligand 12 (CXCL12), a biomarker interfering with tumor angiogenesis in PCa, can be produced by specific cell types identified through bulk RNA-seq, scRNA-seq, and functional assays. Chen et al. [10] performed scRNA-seq on 13 samples from 12 PCa patients and found that active communication between activated endothelial cells promoted PCa invasion; they also provided evidence linking ectopic *KLK3* expression to PCa micrometastasis at the single-cell level, providing support for the underlying therapeutic value of *KLK3* beyond its diagnostic role as a PSA expression product [10]. Therefore, the combination of scRNA-seq and other single-cell analysis technologies provides more therapeutic possibilities for PCa patients from a TME perspective.

Furthermore, accurately identifying patients who exhibit a favorable response to treatment is of utmost importance. Taavitsainen et al. [144] utilized scRNA-seq to determine distinct cellular and transcriptional signatures, leading to the identification of two clusters “PROSGenesis” (derived from prostate cells after ADT treatment) and “Persist” (related to persistent cells during enzalutamide resistance development). These clusters were considered stratified molecular predictors of treatment response. Zhao et al. [111] on the other hand, employed PAM50 to classify PCa cells into two types and found that patients with luminal B tumors showed a poor prognosis but better response to postoperative ADT compared to those with non-luminal B tumors, suggesting the more accurate use of postoperative ADT. Furthermore, numerous novel drugs are being developed based on the specific signatures detected by scRNA-seq. For example, several *HPN*-targeting inhibitors have been designed to enhance PCa treatment [145, 146].

### scRNA-seq analysis is a powerful tool for predicting and monitoring PCa prognosis

The emergence of drug resistance with ADT and second-generation AR antagonists poses a significant threat to patient survival. Previous studies have confirmed the reliability of scRNA-seq as a tool for generating resistance mechanisms and predicting prognosis. Firstly, single-cell data aids in unraveling the underlying mechanisms of drug resistance. Linder et al. [147] integrated single-cell multi-omics data (epigenomics, genomics, transcriptomics, and proteomics) from PCa patients who were either untreated or treated with enzalutamide monotherapy for 3 months, identifying aryl hydrocarbon receptor nuclear translocator-like protein 1, a core component of the circadian rhythm, as a key gene driving enzalutamide-induced *FOXA1* reprogramming and escape from *AR* blockade in PCs cells. Taavitsainen et al. [144] utilized scATAC-seq and scRNA-seq to link chromatin structure changes induced by continuous enzalutamide exposure to transcriptional reprogramming in a preclinical model of enzalutamide-resistant PCa. Transcriptomic reprogramming has also been consistently observed in mouse models by scRNA-seq, potentially associated with ADT resistance and metastasis [96]. From a clinical perspective, scRNA-seq is an ideal strategy for detecting drug resistance. Schnepp et al. [148] employed a combination of scRNA-seq and network analysis to modulate the development of docetaxel resistance and identify potential agents capable of disrupting the resistance networks. They confirmed that trichostatin A is an adjuvant that can disrupt docetaxel resistance, thereby emphasizing the significance of analyzing resistance by network analysis on single-cell transcriptomic data [148]. In addition, the subtype “aggressive variant PCa (AVPC)” was attributed to its drug resistance profile and poor prognosis due to the loss of some tumor suppressor genes [149, 150]. Using single-CTC genomic analysis, Malihi et al. [151] found a significant association between genomic instability in CTC and aggressiveness in advanced PCa, aiding in identifying potential AVPCs. Furthermore, scRNA-seq of PCa CTCs revealed heterogeneity in signaling pathways that may lead to treatment failure. Miyamoto et al. [152] suggested that ectopic expression of Wnt5a attenuates the anti-proliferative effect caused by AR inhibitors. Other studies have also confirmed the predictive value of scRNA-seq for CTCs in PCa metastasis [22, 153]. Barkley et al. [82] proposed a framework based on pan-cancer scRNA-seq data elucidating interactions between PCa cell state and TME, supporting strategies targeting immune evasion, drug resistance, and metastasis. Using similar methods, Cao et al. [154] reported that measuring cell type-specific total mRNA expression can predict PCa phenotypes and clinical outcomes. Table 2 summarizes the clinical application studies utilizing scRNA-seq in PCa [10, 82, 96, 117, 121, 126, 127, 133, 134, 139, 141, 143, 144, 148, 153].

**Table 2:** Summary of studies about clinical applications of scRNA-seq in PCa

Author	Technique	Objective/Conclusion
Cheng et al. [126]	scRNA-seq	CRPC-like cells are present early in the development of PCa and are not exclusively the result of acquired evolutionary selection during androgen deprivation therapy. The lethal CRPC and SCNC phenotypes should be targeted earlier in the disease course of patients with PCa
Karthaus et al. [96]	scRNA-seq	Prostate regeneration is driven by nearly all persisting luminal cells, not just by rare stem cells
He et al. [121]	scRNA-seq	The transcriptional characterization of cancer and immune cells from human mCRPC provides a basis for the development of therapeutic approaches complementing androgen signaling inhibition
Wang et al. [133]	scRNA-seq	Defining the complex expression profiles and advancing the understanding of the genetic and transcriptomic mechanisms leading to NEPC differentiation
Ge et al. [134]	scRNA-seq	Analysis of subclonal and transcriptional heterogeneity and its implication for patient prognosis
Peng et al. [139]	scRNA-seq	Identifying EP4 as a specific target for PCa immunotherapy and demonstrating that YY001 inhibited the growth of prostate tumors by regulating the immune microenvironment and strongly synergized with anti-PD-1 antibodies to convert completely unresponsive PCa into responsive cancers, resulting in marked tumor regression, long-term survival, and lasting immunologic memory
Masetti et al. [141]	scRNA-seq	Identifying a specific TAM subset associated with poor prognosis and recognizing the characteristic transcriptional dysregulation target of lipid metabolism “Marco”
Heidegger et al. [143]	scRNA-seq	Identifying novel PCa TEC targets and highlights CXCR4/CXCL12 interaction as a potential novel target to interfere with tumor angiogenesis in PCa
Chen et al. [10]	scRNA-seq	aECs are enriched in CRPC and promote cancer cell invasion
Schnepp et al. [148]	scRNA-seq	A shared TF activity network drives docetaxel resistance in PCa
Lohr et al. [153]	scRNA-seq	An integrated process to isolate, qualify, and sequence whole exomes of CTCs with high fidelity using a census-based sequencing strategy
Barkley et al. [82]	scRNA-seq; Spatial transcriptomics	Providing a framework for studying how cancer cell states interact with the tumor microenvironment to form organized systems capable of immune evasion, drug resistance, and metastasis
Taavitsainen et al. [144]	scRNA-seq; scATAC-seq	Defining changes in chromatin and gene expression in single-cell populations from pre-clinical models can reveal unrecognized molecular predictors of treatment response
Qiu et al. [127]	scRNA-seq; ChIP-seq	MYC overexpression antagonizes the canonical AR transcriptional program and contributes to prostate tumor initiation and progression by disrupting transcriptional pause release at AR-regulated genes
Rivello et al. [117]	MA-Chip; scRNA-seq	Single-cell extracellular pH measurement for the detection and isolation of highly metabolically active cells (hm-cells) from the tumor microenvironment

*PCa* prostate cancer, *scRNA-seq* single-cell RNA sequencing, *CRPC* castration-resistant PCa, *SCNC* small cell neuroendocrine carcinoma, *mCRPC* metastatic CRPC, *NEPC* neuroendocrine PCa, *PD-1* programmed cell death protein 1, *TAM* tumor-associated macrophage, *aECs* activated endothelial cells, *TEC* tumor endothelial cells, *TF* transcription factor, *CTCs* circulating tumor cells, *MYC* MYC proto-oncogene, *AR* androgen receptor

In conclusion, the applications of scRNA-seq in preclinical models or clinical samples have significantly influenced fundamental research and clinical decision-making, including subsequent advancements in PCa research and the reclassification of PCa, prediction of treatment response, and precise selection of treatment strategies for PCa patients.

## Applications of other single-cell technologies in prostate cancer

In addition to scRNA-seq analysis, other single-cell technologies hold immense potential for exploring the fundamental and clinical applications of PCa. For example, multiple immunofluorescence single-cell spatial imaging and gene expression profiling can help distinguish PCa tissues and achieve precise diagnosis [16]. Moreover, a recent study by Champagne et al. [155] proposed an innovative bioluminescence microscopy approach to explore and evaluate the response to AR-axis-targeted therapy at the single-cell level, demonstrating the sensitivity of the cell population. Live single-cell imaging phenotypes and other assays for quantifying single-cell populations may also be used to develop therapeutic responses and determine optimal treatment plans for different PCa cells. For example, future studies should confirm that LNCap-like cells are sensitive to ADT treatment, whereas C4-2-like cells respond better when ADT is combined with docetaxel, suggesting proper therapeutic strategy. Effective models using single-cell data and drug response information not only enhance machine learning capabilities in predicting cellular drug therapy outcomes but also significantly contribute to drug discovery for specific cancer subgroups and treatments [156]. Molecular probes are another technique applicable at the single-cell level in PCa and hold great promise in the early diagnosis of aggressive PCa and treatment processes. The near-infrared probe developed by Osada et al. [157] combines drugs with heat shock protein 90 (HSP90, a small molecule known for its ability to induce growth and invasion of PCa [158]), resulting in the formation of a novel probe called “HS196”. Subsequent scRNA-seq analysis showed that HS196 was taken up by malignant epithelium in the human prostate, indicating its potential utility in diagnosing PCa and detecting different subtypes [158]. Moreover, a recently reported probe named “semiconducting polymer nanoparticles of 2-[3-(1,3-dicarboxypropyl) ureido] pentanedioic acid (DUPA)-conjugated ligand and bis-isoindigo-based polymer (BTII) (BTII-DUPA SPN)” [19], has been validated for the early detection of PCa [159, 160]. Additionally, owing to the unique anatomical position of the prostate, urine could serve as an innovative, noninvasive, and convenient method for collecting PCa cells to detect potential therapeutic responses. Single-cell proteomics also plays a crucial role in precision medicine for PCa. Karabacak et al. [161] used a single-cell proteomics-based single-cell mass cytometry analysis technology to detect the kinase activity of PTEN-deficient PCa xenograft models and found changes in tumor cell kinase activity as well as drug sensitivity at different sites. It has also been reported that activation of the kinase network induced by microenvironment or cellular states can contribute to heterogeneity and drug sensitivity across different metastatic sites and PCa populations. The selection of the optimal treatment strategy for patients with metastatic PCa should prioritize combination therapy and precise dosages to address all therapeutic vulnerabilities of PCa cells, whether primary or metastatic, in different tissues and organs. In addition, the label-free droplet-based microfluidic approach known as single-cell MA-Chip can predict poor prognosis and therapeutic responses by measuring the extracellular pH and high metabolic state of a single cell [117]. Furthermore, single-cell analysis is helpful in surgical operations. With the assistance of transient absorption microscopy and photoacoustic tomography, the BTII-DUPA SPN has great potential for determining precise surgical margins [19], similar to those achieved by the HS196 probe. Due to its rapid absorption by malignant cells and prolonged retention time, HS196 enables routine detection of PCa as well as intraoperative detection [157]. Remarkably, photodynamic therapy has made remarkable advancements in ablating PCa through targeted laser application at specific wavelengths following systemic administration of a photosensitizer [162, 163]. Thus, substituting the near-infrared molecules in HS196 with photosensitive molecules may provide a more precise treatment approach for PCa, especially for lesions with a high malignant risk. Furthermore, the development of therapeutic drug molecules that bind HSP90 will pave the way for more precise treatments. Physical interaction cell sequencing (PIC-seq) is a novel technique that combines cell sorting of PICs with scRNA-seq using gentle tissue dissociation methods to preserve cell aggregate structure in the tissues [164]. Compared to scRNA-seq, PIC-seq can directly study functional communication between cells involved in physical interactions. This approach better reveals the tumor microenvironment heterogeneity while accurately capturing cell clusters engaged in physical interactions [164, 165]. Although the technology has not yet been widely available, its great potential in cancer research suggests that PIC-seq may provide novel insights into basic and clinical investigates, thereby facilitating the advancement of precision treatment for PCa treatment.

## Conclusions

Due to the scarcity of genome-wide lineages associated with PCa, single-cell analysis and related techniques are increasingly indispensable tools for studying cancer initiation, progression, therapy response, and drug resistance. Despite facing challenges such as low RNA input, amplification bias, batch effect, noise during library preparation and data analysis in scRNA-seq for PCa research, as well as specific issues related to precise sampling of prostates, scRNA-seq provides novel insights for scientific research and clinical management. This technology allows for a more detailed understanding of the disease at an individual patient level by tailoring treatment based on each patient’s unique genetic profile. Such personalized treatment approaches can significantly enhance therapeutic efficacy while reducing side effects. Moreover, scRNA-seq accurately diagnoses PCa by detecting specific molecular markers on the surface of single cells, identifying therapeutic targets and drug resistance mechanisms specifically relevant to this disease, thereby promoting the development of new therapeutic drugs. Integrating multiple-omics analyses with scRNA-seq and other single-cell techniques can address challenges beyond the scope of scRNA-seq alone. These include determining precise surgical margins, identifying drug resistance mechanisms in CRPC, discovering optimal treatments, and providing insights into immunotherapeutic strategies for PCa. Over time, advancements in single-cell data and technologies will contribute significantly to personalized medicine through novel combinations with spatiotemporal transcriptomics. Therefore, it is critical to integrate single-cell data from various models and laboratories to establish cell maps specific to PCa so that single-cell analysis can be better applied to basic research settings while aiding clinical decision-making processes. Ethically, these technologies involve the handling of sensitive genetic information and therefore give rise to privacy concerns. Equitable access to these advanced treatments is also an issue because they may be expensive and not accessible to all social classes. The primary challenge in this rapidly evolving field is to strike a balance between the benefits of these technologies and ethical considerations while ensuring fair access. In terms of data sharing, single-cell data holds immense value for research but also raises concerns about privacy and consent. Sharing such detailed biological data requires robust frameworks that guarantee secure and ethical utilization of the data while maintaining a balance between scientific progress and individual rights. With the establishment of multiple scRNA-seq platforms and technological innovations, lower costs have expanded the market for scRNA-seq applications. The focus of future research lies in combining scRNA-seq with various sequencing methods (such as spatial transcriptomics, ACAT-seq, and ChIP-seq) to explore the pathogenesis, development, and diverse biological processes of PCa at a deeper level. Furthermore, by integrating single-cell technologies with emerging tools, insights into biological complexity at an unprecedented resolution are made possible. These technologies enable studying cellular heterogeneity and comprehending the behavior of individual cells within a complex tissue or organism. Advanced computational methods along with artificial intelligence are increasingly being integrated to manage and interpret vast amounts of generated data effectively. Innovations like high-throughput screening and CRISPR gene editing are being adopted for single-cell analysis, allowing for more precise genomic editing as well as functional studies at the single-cell level. The integration of single-cell technologies with emerging tools is revolutionizing the field of biomedical research, thereby expanding our understanding of disease mechanisms, and paving the way for breakthroughs in personalized medicine.

**Fig. 4:**
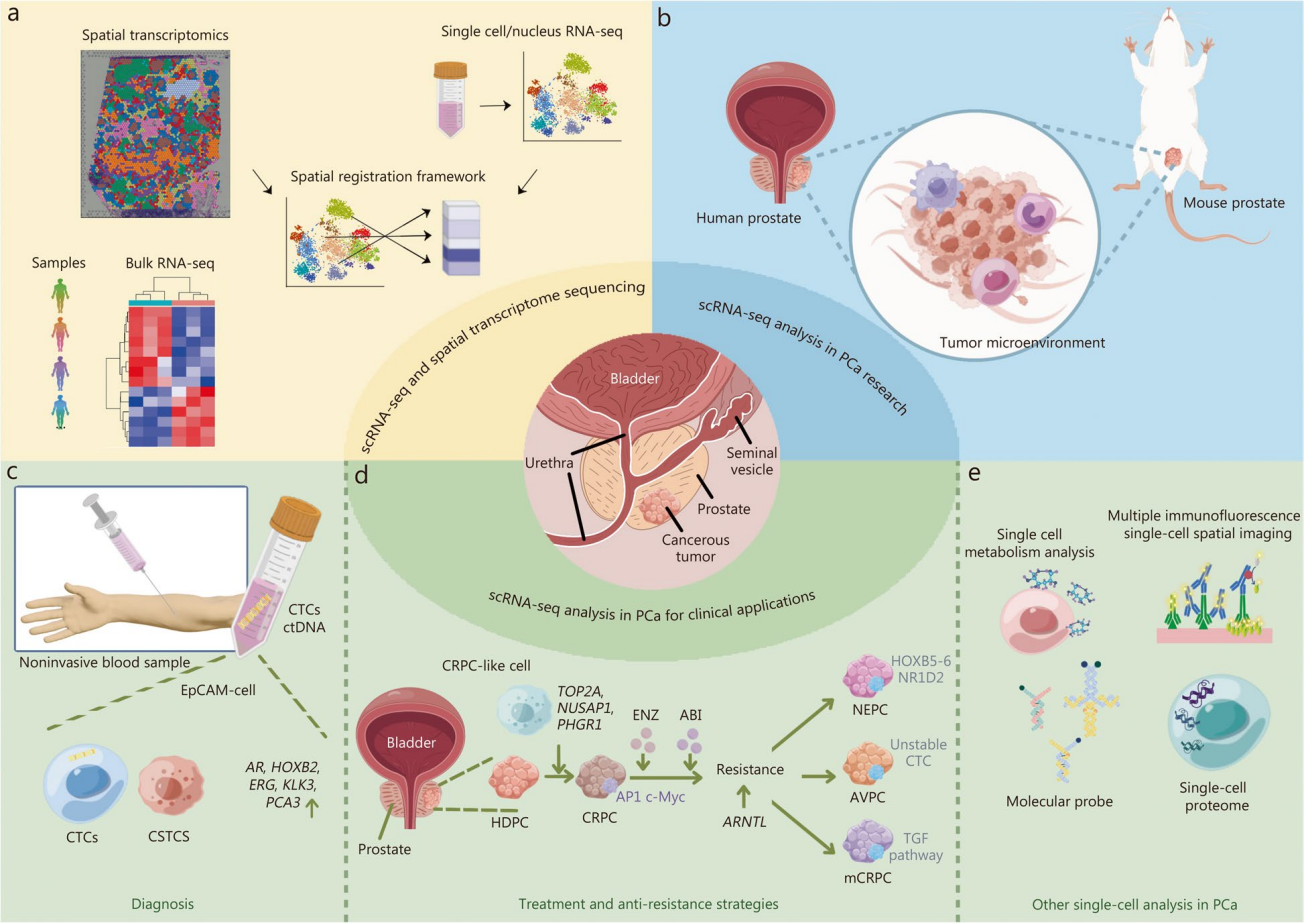
Summary of the contents in this review. **a** scRNA-seq and spatial transcriptome sequencing. **b** scRNA-seq analysis in PCa research, containing human and mouse PCa models, which can help researchers explore the heterogeneity of the tumor microenvironment. **c** scRNA-seq analysis in PCa for clinical diagnosis. Detection of CTCs and ctDNA is a non-invasive method for tumor assessment. There are genes significantly expressed in EpCAM-negative cells in the blood, such as AR, erythroblast transformation-specific (ETS)-related gene, HOXB2, KLK3, and PCA3, helping in identifying either EpCAM-negative CTCs or EpCAM-negative circulating stromal cells. **d** scRNA-seq analysis in PCa for clinical treatment and anti-resistance strategies. HDPC is normally the initial stage of PCa. This type relies on the presence of androgens to grow. Androgen deprivation therapy is a common treatment, including ENZ, ABI and so on. However, over time, some HDPC may develop resistance to hormone therapy and progress to a more aggressive form known as CRPC. In this process, scRNA-seq revealed the presence of highly adaptive CRPC-like cells in the early stages of PCa. These cells continuous to clone and amplify throughout the disease progression. The markers of CRPC-like cells include TOP2A, NUSAP1, PHGR1 and so on. In addition, combining scRNA-seq with other techniques such as ChIP-seq, several important transcription factors have been found to be involved in the progression of drug resistance in PCa, such as AP1, c-Myc and ARNTL. NEPC is a rare and aggressive subtype usually occurring in later stages. NEPC is often associated with resistance to standard treatments for PCa. Treatment options are limited, and prognosis is poor. Through scRNA-seq, some important genes and pathways such as HOXB5, HOXB6, NR1D2 and TGF pathway being identified, which can potentially serve as effective therapeutic targets for subsequent research in mCRPC and NEPC. **e** Other single-cell analysis in PCa, including single cell metabolism analysis, multiple immunofluorescence single-cell sptial imaging, molecular probe, single-cell proteome and so on. scRNA-seq single-cell RNA sequencing, PCa prostate cancer, CTCs circulating tumor cells, ctDNA circulating tumor DNA, EpCAM epithelial cell adhesion molecule, CSTCS circulating stromal cells, AR androgen receptor, HOXB2 Homeobox B2, ERG erythroblast transformation-specific (ETS)-related gene, KLK3 kallikrein-3, PCA3 prostate cancer associated 3, CRPC castration-resistant prostate cancer, HDPC hormone dependent prostate cancer, ENZ enzalutamide, ABI Abiraterone, NEPC neuroendocrine prostate cancer, mCRPC metastatic castration-resistant prostate cancer, AVPC aggressive-variant prostate cancer, TOP2A DNA topoisomerase II alpha, NUSAP1 nucleolar and spindle associated protein 1, PHGR1 proline, histidine and glycine rich 1, ChIP-seq chromatin immunoprecipitation followed by sequencing, AP1 activator protein 1, c-Myc myelocytomatosis oncogene, ARNTL aryl hydrocarbon receptor nuclear translocator like, HOXB5 homeobox B5, HOXB6 homeobox B6, NR1D2 nuclear receptor subfamily 1 group D member 2, TGF transforming growth factor

In summary, in this review, we first discussed recent advancements in single-cell and spatial transcriptomics, focusing on the integration of single-cell and spatiotemporal transcriptomics. Then we focused on the application of scRNA-seq in PCa research. We outlined the differences between mouse and human prostate cancer revealed by scRNA-seq studies and discussed the application of multi-omics methods involving scRNA-seq to identify molecular targets for diagnosis, treatment, and drug resistance in prostate cancer. At last, we explored the potential of other single-cell technologies in developing immunotherapy and other innovative treatment strategies for CRPC, as well as their broader clinical applications in precision medicine. We summarized the main contents of this review in Fig. 4.

## Data Availability

Not applicable.

## Abbreviations

ADT: Androgen deprivation therapy
ChIP-seq: Chromatin immunoprecipitation followed by sequencing
CRPC: Castration-resistant PCa
CTCs: Circulating tumor cells
EpCAM: Epithelial cell adhesion molecule
HVGs: Highly variable genes
LP: Lateral lobes
mCRPC: Metastatic castration-resistant PCa
NEPC: Neuroendocrine carcinoma
PCa: Prostate cancer
PSA: Prostate-specific antigen
QC: Quality control
TAMs: Tumor-associated macrophages
TME: Tumor microenvironment
UMI: Unique molecular identifier
